# Heterologous Boost Following *Mycobacterium bovis BCG* Reduces the Late Persistent, Rather Than the Early Stage of Intranasal Tuberculosis Challenge Infection

**DOI:** 10.3389/fimmu.2018.02439

**Published:** 2018-10-30

**Authors:** Yaqi Wu, Ming Cai, Jilei Ma, Xindong Teng, Maopeng Tian, Eman Borham Mohamed Borham Bassuoney, Xionglin Fan

**Affiliations:** ^1^Department of Pathogen Biology, School of Basic Medicine, Tongji Medical College, Huazhong University of Science and Technology, Wuhan, China; ^2^Department of Gastrointestinal Surgery, Union Hospital, Tongji Medical College, Huazhong University of Science and Technology, Wuhan, China; ^3^Shandong International Travel Healthcare Center, Shandong Entry-Exit Inspection and Quarantine Bureau, Qingdao, China

**Keywords:** BCG, heterologous boost, memory T cells, early stage of tuberculosis, late persistent tuberculosis

## Abstract

Adults are the leading population affected by tuberculosis (TB) epidemic and death. Developing an effective vaccine against adult TB is urgently needed. *Mycobacterium bovis* Bacillus Calmette-Guerin (BCG) prime-heterologous boost strategy has been explored extensively to protect adults against primary TB infection, but the majority of experimental regimens have not improved the protection primed by the BCG vaccine. The reason attributed to the failure remains unknown. In this study, CTT3H-based vaccines, namely DMT adjuvanted CTT3H subunit or DNA vaccine (pCTT3H-DMT), and recombinant adenovirus rAdCTT3H were constructed. Protective efficacy and immunogenicity of BCG prime-CTT3H based boosters were compared in C57BL/c mice models of primary or late persistent TB infection. Similar protective efficacy against early intranasal infection was provided by different CTT3H-based vaccines alone in vaccinated mice, and their protection was inferior to that of the BCG vaccine. In addition, CTT3H-based heterologous boosters did not enhance the protection conferred by the BCG vaccine against primary infection. However, all of these three boosters provided stronger protection against late persistent TB infection than BCG alone, regardless of vaccine types. Although BCG prime-boosters elicited Th1-biased responses to the antigen CTT3H, the number of CTT3H-sepcific IFN-γ-expressing T_EM_ (CD62L^lo^CD44^hi^) and IL-2-expressing T_CM_ (CD62L^hi^CD44^hi^) cells in the spleen was not improved before exposure to *Mycobacterium tuberculosis* infection. In contrast, IFN-γ^+^ T_EM_ and IL-2^+^ T_CM_ cells in spleens, especially in lungs were significantly increased in BCG prime-boosters after exposure vaccination. Our results indicate that BCG prime-boost strategy might be a promising measure for the prevention against late persistent TB infection by induction of IFN-γ^+^ T_EM_ and IL-2^+^ T_CM_ cells in the lung, which can be used as alternative biomarkers for guiding the clinical practice and future development of TB vaccine for adults.

## Introduction

Despite significant progress in the pipeline for new diagnostics, drugs, and vaccine candidates, tuberculosis (TB) remains one of the deadliest killers among infectious diseases (1). As the only licensed vaccine for TB, *Mycobacterium bovis* Bacillus Calmette-Guerin (BCG) is recommended to vaccinate infants worldwide and can provide effective protection against several serious forms of TB in children even though its protection wanes with time and only lasts 10–15 years (2). Accordingly, BCG vaccine cannot control the prevalence and transmission of adult, mostly pulmonary TB. As estimated by WHO in 2017, there were ~ 10 million new TB cases, and 90% occurred in adults with only 10% in children (1). Furthermore, one-third of the world population is estimated to have latent TB infection (LTBI), of which 5–10% would progress to active TB during their lifetime (3). Therefore, there is an urgent need to develop an effective vaccine against adult TB.

Adult TB is primarily caused by endogenous reactivation of LTBI secondarily by exogenous contagion or reinfection. Vaccine candidates such as DNA and subunit vaccines, recombinant viral vectors, genetically modified BCG, or attenuated *Mycobacterium tuberculosis* strains have been explored extensively for adult TB (4). However, it remains elusive to replace the BCG vaccine because few vaccine candidates can provide vastly superior protection than the BCG vaccine in vaccinated animals (5). Furthermore, extensive research has been carried out on the heterologous boost strategy, aimed to extend the protection period primed by the BCG vaccine (5, 6). Unfortunately, almost half of heterologous boost regimens could not demonstrate better efficacy than the BCG vaccine alone against primary *M. tuberculosis* infection in various animal models, such as mice, guinea pigs, cattle, and non-human primates (5, 6). BCG prime and a modified vaccinia virus Ankara expressing antigen 85A (MVA85A) boost regimen might elicit a stronger Th1-typed immune response than the BCG vaccine alone, but MVA85A boosted BCG-primed infants did not show any improved efficacy against TB in a clinical trial (7). The reasons attributed to these failures remain unknown, thus significantly hindering the development of BCG prime-boost strategy.

Recently, we reported that a regimen of BCG prime and DMT (DDA-MPLA-TDB) adjuvanted multistage subunit protein WH121 showed a stronger ability to protect mice against *M. tuberculosis* post-exposure infection than BCG alone or BCG repeat vaccination in mice (8). Furthermore, subunit vaccine CMFO in adjuvant of DMT enhanced the BCG vaccine to thwart the reactivation of LTBI (9). Therefore, whether heterologous boost regimens are more efficacious to prevent against LTBI than primary infection remains to be investigated. CTT3H consists of five antigens of *M. tuberculosis* containing CD8^+^ epitopes: CFP10, TB10.4, TB8.4, Rv3165c, and HBHA (10). In addition, CFP10 and TB10.4-specific cytolytic CD8^+^T cells were found in the lung of mice as early as 3 weeks after infection (11). Besides CD8^+^ T cells, Rv3165c induced specific-CD4^+^T cells both in active TB patients and LTBI individuals (12). HBHA, expressed by both *M. tuberculosis* and BCG, is an adhesion molecule mediating adherence to epithelial cells and plays an important role in extrapulmonary dissemination (13). In this study, CTT3H was chosen to develop recombinant adenovirus vector, the adjuvant DMT emulsified DNA or protein subunit vaccines in this study. Differential protective efficacy of three vaccines against primary *M. tuberculosis* infection was compared with BCG in vaccinated C57BL/6 mice. More importantly, the immunogenicity and protection of BCG prime-boost regimens against primary infection and late persistent infection were compared and assessed in mouse models.

## Materials and methods

### Construction and identification of recombinant adenovirus rAdCTT3H and eukaryotic expression plasmid pCTT3H

Recombinant plasmid pDC316-CTT3H, composed of the tandem-fusion of genes encoding the signal peptide of human tissue plasminogen activator (SP_tPA_) and the fusion protein CTT3H, was commercially synthesized (Life Technologies) (10). A recombinant replication-deficient adenovirus rAdCTT3H was packaged and constructed by co-transfecting plasmids pDC316-CTT3H and pBHGloxΔE1, 3 Cre into human embryonic kidney 293 (HEK293, ATCC® CRL-1573 ™) cells with Lipofactamine 2000 reagent (Invitrogen), as previously described (14). To construct DNA vaccine, the fusion gene was obtained by polymerase chain reaction (PCR) with the specific primers targeting to the plasmid pDC316-CTT3H and subcloned into the eukaryotic expression vector pVAX-1 (Thermo Fisher Scientific), as previously described (15). The resultant construction was named pCTT3H and the inserted gene was verified by enzyme digestion and DNA sequencing.

1 × 10^6^ HEK293T cells (ATCC® CRL-3216 ™) were plated in each well of 6-well plates and cultured at 5% CO_2_, 37°C in Dulbecco's modified Eagle medium (DMEM) (Hyclone), supplemented with 10% fetal bovine serum (FBS) (Hyclone), 100 U/mL penicillin and streptomycin. 24 h later, cells were transfected with the plasmid pCTT3H using Lipofectamine 2000 reagent (Invitrogen). The pVAX1-transfected cells were used as a negative control. Alternatively, cells were infected with either rAdCTT3H or wild-type adenovirus serotype 5 Adnull (Neuron Biotech) at a multiplicity of infection (MOI) of 10 for 48 h. Then, supernatant and cell lysates were collected and prepared, respectively. Fifty micrograms of proteins from either supernatant or cell lysates were analyzed by 12% SDS-PAGE. Western blotting was explored to confirm the expression of the fusion protein CTT3H using mouse anti-CTT3H sera (10) as the primary antibody and peroxidase conjugated goat anti-mouse IgG (Proteintech Biotech) as the secondary antibody. The immunoblots were visualized using a BeyoECL Plus kit (Beyotime Biotech).

**Figure 1:**
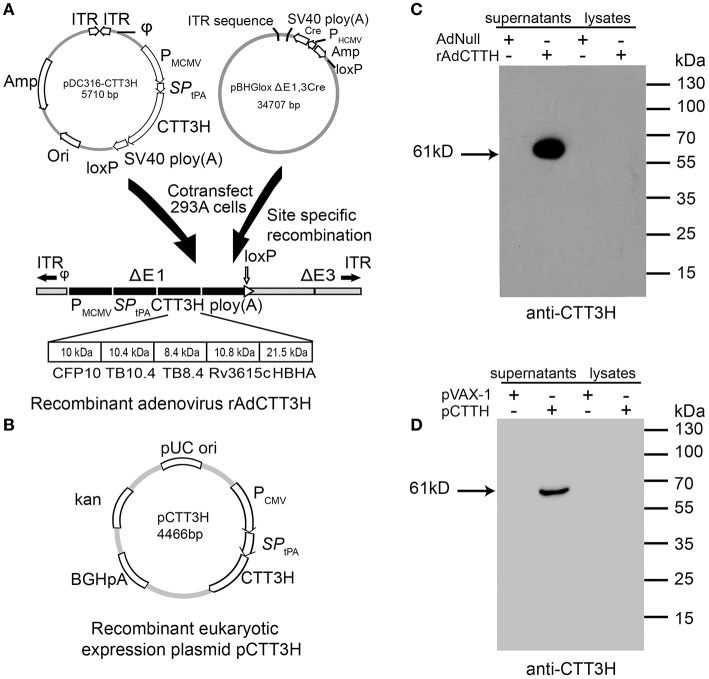
Construction and identification of rAdCTT3H and pCTT3H. **(A)** The structural diagram of recombinant replication-deficient adenovirus serotype 5-based rAdCTT3H. **(B)** The structural diagram of the recombinant eukaryotic expression plasmid pCTT3H. Identification of the recombinant protein CTT3H expressed in the supernatant or lysates of rAdCTT3H infected HEK293T cells **(C)** or pCTT3H transfected HEK293T cells **(D)** by western blotting with anti-CTT3H antibody.

Both recombinant replication-deficient adenovirus serotype 5-based rAdCTT3H and recombinant eukaryotic expressing plasmid pCTT3H were successfully constructed (Figures 1A,B). Secretion of the recombinant fusion protein CTT3H with a size of about 61kDa was detected only in supernatants rather than cellular lysates generated from the rAdCTT3H infected- or the plasmid pCTT3H transfected- HEK293T cells, as confirmed by western blotting with anti-CTT3H mouse polyclonal antibody (Figures 1C,D). In contrast, no specific expression in culture supernatants or cellular lysates from Adnull or pVAX-1 treated HEK293T cells was detected, respectively (Figures 1C,D).

### Preparation of vaccines

The purification and quantitation of recombinant adenovirus rAdCTT3H, recombinant protein CTT3H, and recombinant eukaryotic plasmid pCTT3H was performed as previously described, respectively (10, 14, 15). The concentration of the endotoxin in each purified products was detected by ToxinSensorTM Chromogenic LAL Endotoxin Assay Kit (Genscript). The adjuvant DMT liposome was prepared by lipid film hydration method and rehydration in 10 mM sterile Tris-buffer (pH 7.4) as previously described (10). DMT adjuvanted subunit vaccine CTT3H-DMT and DNA vaccine pCTT3H-DMT were prepared by emulsifying 100 μL of the DMT liposome and 100 μL of either protein CTT3H (50 μg/100 μL) or plasmid pCTT3H solution (50 μg/100 μL), respectively.

### Mice and immunization

**Figure 2:**
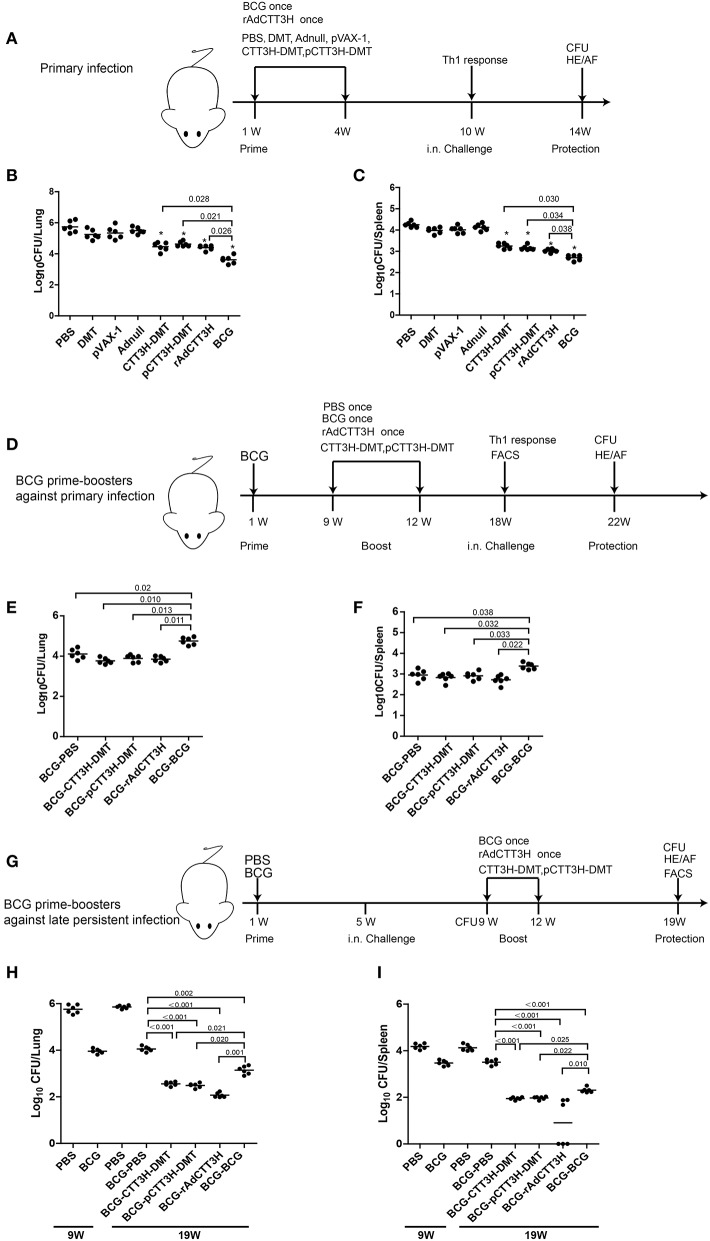
Comparison of protective efficacy among different regimens. **(A)** The immunization and challenge schedules of three CTT3H-based vaccines alone immunized C57BL/6 mice against primary *M. tuberculosis* infection (*n* = 6). C57BL/6 mice were immunized with CTT3H-DMT, pCTT3H-DMT, and rAdCTT3H, respectively. BCG was used as positive control. PBS, DMT, pVAX-1, or Adnull were used as negative controls. At the tenth week after immunization, mice were challenged i.n. with approximately 100 CFU virulent *M. tuberculosis* H37Rv strain. Four weeks post-challenge, spleen, and lung were aseptically removed from each mouse and the number of *M. tuberculosis* in both organs was cultured and enumerated. The results are shown as (mean ± SEM) log_10_ CFU/lung **(B)** or spleen **(C)** of different groups. **(D)** The protocol of BCG prime-CTT3H-based boosters in mice against primary infection (*n* = 6). Bacterial load in the lung **(E)** and spleen **(F)** of the different groups were shown as (mean ± SEM) log_10_ CFU/organ. (**G**) The protocol of BCG prime-CTT3H-based boosters against mice late persistent TB infection (*n* = 6). Bacterial load in the lung **(H)** and the spleen **(I)** of different groups was shown as (mean ± SEM) log_10_ CFU/organ. All experiments were repeated twice with similar results.

Specific pathogen-free (SPF) C57BL/6 female mice, aged 6–8 weeks, were purchased from the Center for Animal Experiments of Wuhan University (Wuhan, China). Mice were randomly divided into different groups and were housed on the animal feeding cabinet (VentiRack) in an ABSL-3 laboratory. The immunization regimens were listed in Figure 2. For different regimens, a dose of 200 μL either pCTT3H-DMT or CTT3H-DMT was immunized intramuscularly (i.m.) twice at 3-weeks intervals. rAdCTT3H was immunized intranasally (i.n.) once with a dose of 5 × 10^8^ PFU. 1 × 10^6^ CFU of BCG China in a volume of 200 μL PBS was vaccinated subcutaneously (s.c.) once at the time of the first vaccination and used as a positive control. PBS, DMT, Adnull, or pVAX-1 alone were used as negative controls.

### Challenged with virulent *M. tuberculosis* H37Rv

Mice models of primary infection and late persistent TB infection were established as previously described (8, 9). Vaccinated mice were infected i.n. with about 100 CFU live *M. tuberculosis* H37Rv as mentioned in different regimens (Figure 2). At different time-points post-challenge, mice were sacrificed. Lung and spleen from six mice in each group were aseptically removed, respectively. The bacterial load per organ was enumerated by plating serial dilutions of whole organ homogenates on Middlebrook 7H11 agar. When required, 2 μg/ml of 2-thiophenecarboxylic acid hydrazide (TCH, Beijing Luqiao Corp.) was added to selectively inhibit the growth of the residual BCG. The lung from three mice in each group was fixed in 4% paraformaldehyde solution, embedded in paraffin, sectioned and stained with hematoxylin and eosin (HE) and acid-fast (AF) staining. Lung histopathological scores of three mice in different groups were evaluated as previously described (9).

### CTT3H -specific IgG antibody and subclasses tittered by ELISA

Serum from each mouse in different vaccinated groups was collected and the CTT3H-specific endpoint titers for IgG, IgG1, and IgG2a (Abcam) were tested by ELISA, as previously described (10).

### CTT3H-specific IFN-γ secreted by splenocytes detected by ELISA

2.5 × 10^6^ of splenocytes were seeded into each well of 24-well microtiter plates and incubated with 10 μg/mL of CTT3H protein at 37°C in a humidified CO_2_ incubator for 72 h (10). Supernatants from each well were collected and a commercial mouse IFN-γ ELISA kit (Multi Sciences LTD) was used to measure the concentration of IFN-γ in the supernatants with a detection limit of 5 pg/ml. CTT3H-specifc IFN-γ concentration was calculated by subtracting the background of medium control. The results are showed as the mean ± SD (pg/mL) for each group (*n* = 6).

### *In vivo* measurement of cytotoxic T cells responses to TB10.4

The CFSE method was used to evaluate the CTL effects against TB10.4 (Qiangyao Biotech) of different vaccinated mice as previously described (10) and analyzed by a flow cytometer (Figure S1). The specific rate of killing of *in vivo* CTL assay was calculated by the formula: 1 – (%CFSE high cells/%CFSE low cells) × 100 (10). The results are expressed as the mean ± SEM of six mice per group.

### CTT3H-specific memory T cells detected by intracellular flow cytometry

Total splenocytes or pneumonocytes from each mouse were prepared. These cells were stained for CD4 or CD8, intracellular cytokines IFN-γ and IL-2, and surface markers CD62L and CD44. The absolute number of IFN-γ^+^ and IL-2^+^ T cells, central memory T cells (T_CM_, CD62L^hi^CD44^hi^) and effector memory T cells (T_EM_, CD62L^lo^CD44^hi^) was detected as previously described (9). The results are shown as mean ± SD per group (*n* = 6).

### Statistical analysis

A two-tailed Student's *T*-test was performed for pair-wise comparisons with SPSS 19.0 software (SPSS 19.0, Chicago, IL, USA). One-way ANOVA test was used for analysis, when three or more experimental groups were compared, with Tukey's honestly significant difference (HSD) *post-hoc* test in SPSS 19.0. A value of *p* < 0.05 was considered significant.

## Results

### Enhanced protection against late persistent rather than primary TB infection conferred by heterologous boost BCG with CTT3H-based vaccines

To evaluate the effect of vaccine types on the protection, the protective efficacy against primary *M. tuberculosis* infection between DMT adjuvanted CTT3H subunit protein vaccine, DNA vaccine pCTT3H in adjuvant of DMT, and recombinant adenovirus rAdCTT3H vaccine were firstly compared in different vaccinated mice. At the tenth week after immunization, different vaccinated C57BL/6 mice were challenged i.n. with about 100 CFU of virulent *M. tuberculosis* H37Rv strain. Four weeks after infection, bacterial load in the lung, spleen, and lung histopathology were used to assess vaccine-induced protection (Figure 2A). Among all groups, PBS control mice, as well as Adnull and pVAX-1 groups, had the highest bacterial load in the both lung (Figure 2B) and spleen (Figure 2C) (*p* < 0.05), while the most significant inhibition of the growth of *M. tuberculosis* in both organs was obtained in BCG vaccinated mice (*p* < 0.05). Interestingly, both DMT adjuvanted CTT3H and pCTT3H vaccinated mice conferred much more significant protection than the DMT alone treated mice (*p* < 0.05). rAdCTT3H vaccinated mice also more significantly reduced the bacterial load in the lung and spleen than PBS or Adnull controls (*p* < 0.05). In addition, there was no statistical difference of organ bacterial load among rAdCTT3H, CTT3H-DMT, and pCTT3H-DMT groups.

To further investigate the boost effect and the effect of booster vaccine types on the BCG-primed protection against primary infection, mice were first vaccinated with the BCG vaccine and then boosted with three CTT3H-based vaccines at the ninth week. Nine weeks later, mice were challenged i.n. with about 100 CFU of virulent *M. tuberculosis*. Four weeks post-challenge, protection was evaluated in different groups (Figure 2D). BCG repeat vaccinated mice had the highest bacterial load in both lung (Figure 2E) and spleen (Figure 2F) of all groups. Interestingly, there was no statistical difference of the bacterial load in both lung and spleen between heterogeneous CTT3H boosters and the BCG-PBS control group, respectively. Therefore, our results suggest that different CTT3H-based vaccines could not enhance BCG primed mice to protect over the BCG vaccine alone against primary infection, regardless of vaccine types.

To explore the protective efficacy of heterologous boost strategy against LTBI, BCG primed mice were challenged with almost 100 CFU *M. tuberculosis* H37Rv at the fifth week after immunization (Figure 2G). Four weeks later, about 10^4^ CFU and 10^3.5^ CFU of *M. tuberculosis*, respectively, persisted in the lung and the spleen of BCG-primed mice and remained stable during the whole experimental period (Figures 2H,I). Consistent with the previous report (9), the mouse models of late persistent TB infection to mimic LTBI were established successfully, and then boosted with CTT3H-based vaccines or BCG. At the nineteenth week, bacterial load in both lung and spleen from heterologous boosted mice was decreased more significantly than the BCG-PBS control group (Figures 2H,I). In particular, almost half of rAdCTT3H-boosted mice significantly inhibited the growth of *M. tuberculosis* in their spleens (Figure 2I). Unlike protection against primary infection, repeated BCG vaccination also resulted in a more significant decrease in bacterial load of both lung and spleen than in the BCG-PBS group, even if that protection was inferior to those of heterologous boosters. Taken together, our results demonstrated that different CTT3H-based vaccines, regardless of vaccine types, could enhance BCG primed mice to reduce the bacteria load in the organs from late persistent but early stage of intranasal *M. tuberculosis* infection.

### CTT3H specific Th1- biased responses elicited by CTT3H-based vaccines

**Figure 3:**
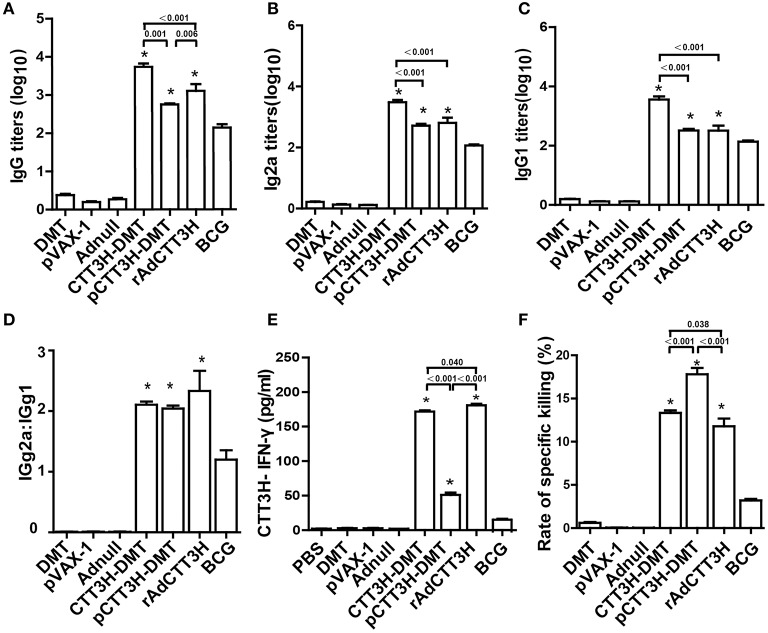
Comparison of CTT3H-specific Th1 responses among three CTT3H-based vaccines immunized C57BL/6 mice (*n* = 6). The immunization schedule was described in Figure 2A. At the tenth week after immunization, sera from each C57BL/6 mice of different groups were collected and titers of anti-CTT3H antibodies IgG **(A)**, IgG2a **(B)**, and IgG1 **(C)** were detected by ELISA. The results are shown as (mean ± SEM) log_10_ endpoint titers. **(D)** The ratio of IgG2a/IgG1 of different vaccinated groups. **(E)** The levels of CTT3H-specific IFN-γ secreted by splenocytes. The concentration of IFN-γ in supernatants was detected by ELISA and the results are expressed as mean ± SD (pg/mL). **(F)** Comparison of CTL response to TB10.4 among different vaccinated groups (mean ± SEM). ^*^p < 0.05 vs. the BCG group. All experiments were repeated twice with similar results.

To assess the immunogenicity of different CTT3H-based vaccines, vaccinated mice were sacrificed for immunological analysis at the tenth week after immunization. PBS, DMT, Adnull, and pVAX-1 treated control mice did not produce any antibodies, including IgG, IgG1, or IgG2a against the recombinant protein CTT3H, as expected (Figure 3). In contrast, higher levels of CTT3H-specific IgG, IgG1, and IgG2a antibodies as well as the higher ratio of IgG2a/IgG1 were induced in mice vaccinated with all three CTT3H-based vaccines vs. BCG (*p* < 0.05) (Figures 3A–D). Among all groups, CTT3H-DMT elicited the highest titers of IgG, IgG1, and IgG2a, while rAdCTT3H vaccinated mice elicited the highest ratio of IgG2a/IgG1. In addition, splenocytes from control mice also did not secrete any INF-γ. Compared to control groups, BCG-vaccinated mice produced much higher levels of both CTT3H specific IFN-γ and CTL response to TB10.4. Both levels in three groups of CTT3H-based vaccinated mice were also much higher than those in the BCG group. Of all groups, the highest concentration of CTT3H specific IFN-γ was secreted by the rAdCTT3H group (Figure 3E), while the strongest CTL response to TB10.4 was detected in pCTT3H-DMT vaccinated mice (Figure 3F).

### Th1 biased response induced by BCG prime-CTT3H-based boosters

**Figure 4:**
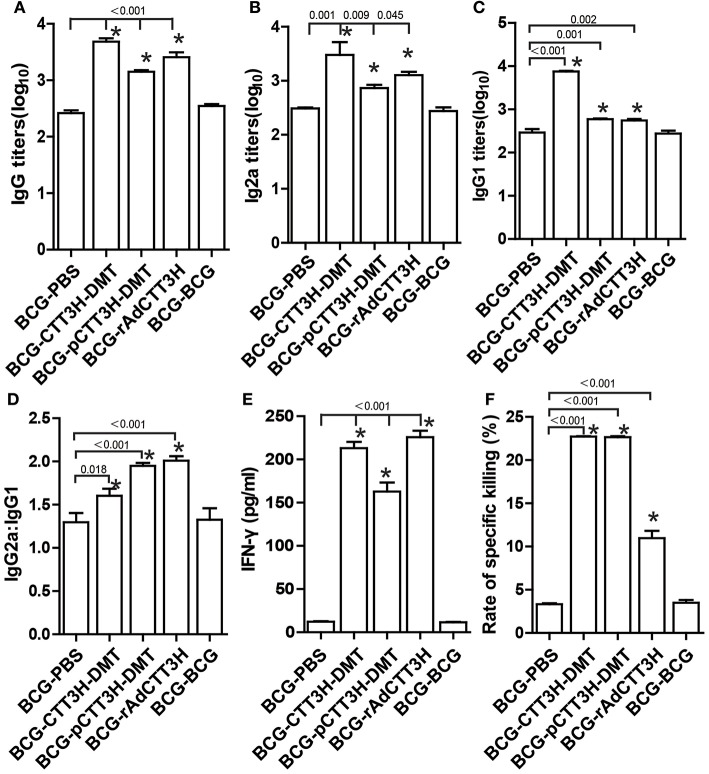
Th1 biased response induced by BCG prime-CTT3H-based boosters (*n* = 6). The immunization schedule was described in Figure 2D. ELISA was used to determine titers of anti-CTT3H IgG **(A)**, IgG2a **(B)**, and IgG1**(C)** in the serum from each C57BL/6 mouse in BCG prime-booster vaccinated groups at the eighteenth week. **(D)** The ratio of IgG2a/IgG1 of different vaccinated groups. **(E)** The levels of CTT3H-specific IFN-γ secreted by splenocytes (mean ± SD pg/mL). **(F)** CTL response to TB10.4 (mean ± SEM). ^*^p < 0.05 vs. the BCG repeated vaccination group. All experiments were repeated twice with similar results.

To investigate the immunological mechanism related with failures of boost strategy against primary infection, Th1-typed response was also analyzed in different vaccinated mice before challenge. Higher levels of anti-CTT3H IgG, IgG1, IgG2a, IgG2a/IgG1, IFN-γ, and CTL responses were induced in mice vaccinated with BCG and CTT3H-based boosters than the BCG-PBS control group (*p* < 0.05) (Figures 4A–D). Among three types of boosters, the highest level of IgG2a/IgG1 and the strongest IFN-γ response were obtained in rAdCTT3H boosted mice (Figures 4D,E), while the most significant CTL response to TB10.4 was detected in DMT adjuvanted DNA or protein boosted mice (Figure 4F). However, BCG repeat vaccination did not enhance any of these immunological biomarkers, compared to the BCG-PBS group.

### IFN-γ^+^ T_EM_ and IL-2^+^ T_CM_ cells decreased in BCG prime-boosters before exposure

**Figure 5:**
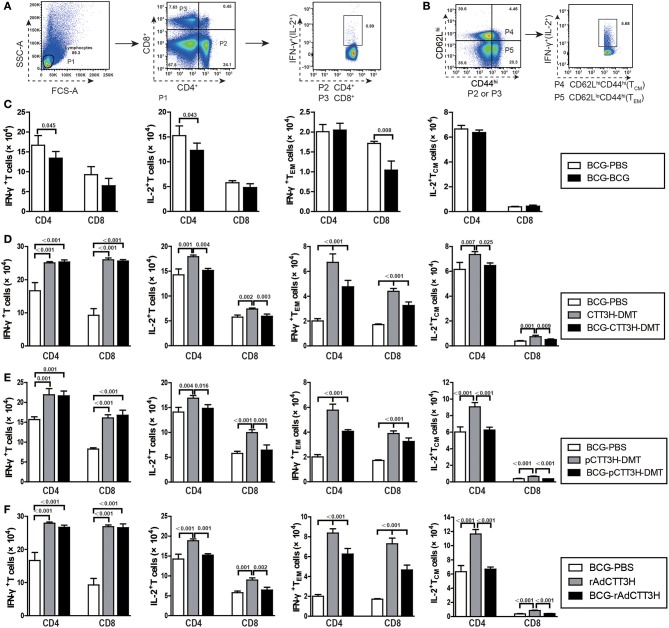
CTT3H antigen-specific T cells in spleens induced by BCG prime-CTT3H-based boosters before exposure (*n* = 6). The immunization protocol was described in Figure 2D. At the eighteenth week, splenocytes from heterologous boosters with PBS, CTT3H-DMT, pCTT3H-DMT, rAdCTT3H, and BCG vaccinated mice were collected and counted. 2.5 × 10^6^ cells were seeded in each well of a 24-well plate and stimulated with CTT3H (10 μg/mL) as described in detail in the section of Materials and Methods. The absolute number of CTT3H-specific IFN-γ^+^ (or IL-2 ^+^) CD4 ^+^ (or CD8 ^+^) T cells, IFN-γ^+^ CD4 ^+^ (or CD8 ^+^) T_EM_ cells, and IL-2^+^ CD4 ^+^ (or CD8 ^+^) T_CM_ cells from spleens were detected by flow cytometry. Results are shown as mean ± SD (*n* = 6). **(A,B)** Flow cytometry analysis strategy, **(C)** comparing BCG prime and BCG repeated boost strategy, **(D,F)** comparing BCG prime, subunit vaccine prime, and BCG prime and boost with subunit vaccine including protein vaccine **(D)**, DNA vaccine **(E)**, and recombinant adenovirus vaccine **(F)**.

IFN-γ^+^ T_EM_ and IL-2^+^ T_CM_ cells in the spleen are thought as effective biomarkers to predict the vaccine-induced early and persistent protection (8). To further establish the immunological mechanism related with the failure of boost strategy against primary infection, the number of CTT3H-specific IFN-γ or IL-2 secreting T cells (Figure 5A), IL-2-expressing T_CM_ (CD62L^hi^CD44^hi^) cells, and IFN-γ-positive T_EM_ (CD62L^lo^CD44^hi^) cells (Figure 5B) in splenocytes between BCG prime-boosted mice was determined. BCG repeat vaccination more significantly decreased the number of IFN-γ^+^ or IL-2^+^ CD4^+^ T cells, and IFN-γ^+^ CD8^+^T_EM_ cells in the spleen vs. BCG-PBS control mice (*p* < 0.05, Figure 5C). Interestingly, BCG prime-heterologous boosters also did not improve the levels of all biomarkers, but resulted in a more significant decrease of the levels of IFN-γ^+^ T_EM_ cells, IL-2^+^ T cells, and IL-2^+^ T_CM_ cells in the spleen, when compared with the respective booster control mice. Compared to the BCG-PBS control group, CTT3H-based boosters only more significantly increased the levels of IFN-γ^+^ T cells and IFN-γ^+^ T_EM_ cells in the spleen (Figures 5D–F), regardless of vaccine types (Figure S2).

### IFN-γ^+^ T_em_ and IL-2^+^ T_CM_ cells increased in the spleen of BCG prime-boosters after exposure

**Figure 6:**
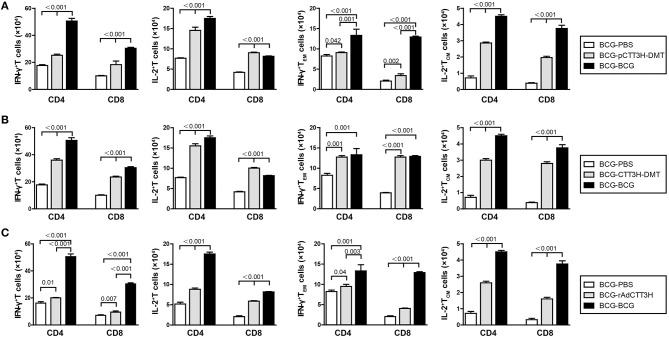
CTT3H antigen-specific T cells in spleens induced by BCG prime-CTT3H-based boosters after exposure (*n* = 6). The immunization and infection protocols were described in Figure 2G. At the nineteenth week, the absolute number of CTT3H-specific IFN-γ^+^ (or IL-2 ^+^) CD4 ^+^ (or CD8 ^+^) T cells, IFN-γ^+^ CD4 ^+^ (or CD8 ^+^) T_EM_ cells, and IL-2^+^ CD4 ^+^ (or CD8 ^+^) T_CM_ cells from spleens were detected by flow cytometry. Results are shown as mean ± SD (*n* = 6). **(A–C)** Comparing BCG prime, BCG repeated boosting, and BCG prime and boosted with subunit vaccine strategies including DNA vaccine **(A)**, protein vaccine **(B)**, and recombinant adenovirus vaccine **(C)**.

To investigate immunological effects related with the protection against late persistent TB infection, IFN-γ^+^ or IL-2^+^ T cells, and memory T cells in the spleen were further analyzed 14 weeks post-challenge (Figure 6). Among all the treatment, repeat BCG vaccination induced the most significant increase in the number of IFN-γ^+^ or IL-2^+^ T cells, IFN-γ^+^ T_EM_ and IL-2^+^ T_CM_ cells in the spleen, except the levels of IL-2^+^ CD8^+^ T cells in both DMT adjuvanted boosters and IFN-γ^+^ T_EM_ in CTT3H- DMT boost group (*p* < 0.05, Figure 6). Regardless of vaccine types (Figure S3A), heterologous boosters with CTT3H-based vaccines induced a more significant increase of all these biomarkers than BCG-PBS control group (**Figure 8**). In particular, IFN-γ^+^ ${\text{CD}}_{4}^{+}$ T cells and IFN-γ^+^ ${\text{CD}}_{4}^{+}$T_EM_ cells were dominated in the spleen of all BCG prime-CTT3H boost groups.

### IL-2^+^ T_CM_ and IFN-γ^+^ T_EM_ in the lung increased in BCG prime-boosters after exposure

**Figure 7:**
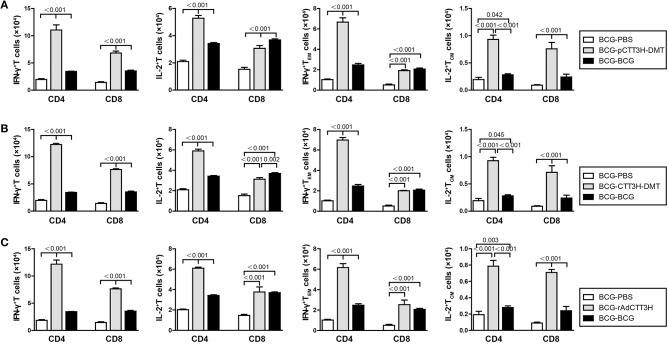
CTT3H antigen-specific T cells in lungs induced by BCG prime-CTT3H-based boosters after exposure (*n* = 6). The immunization and infection protocols were described in Figure 2G. At the nineteenth week, the absolute number of CTT3H-specific IFN-γ^+^ (or IL-2 ^+^) CD4 ^+^ (or CD8 ^+^) T cells, IFN-γ^+^ CD4 ^+^ (or CD8 ^+^) T_EM_ cells, and IL-2^+^ CD4 ^+^ (or CD8 ^+^) T_CM_ cells in pneumonocytes were detected by flow cytometry. Results are shown as mean ± SD (*n* = 6). **(A–C)** Comparing BCG prime, BCG repeated boosting, and BCG prime and boosted with subunit vaccine strategies including DNA vaccine **(A)**, protein vaccine **(B)**, and recombinant adenovirus vaccine **(C)**.

Furthermore, sterilizing immunity is attributed to drive IFN-γ^+^ T_EM_ and IL-2^+^ T_CM_ cells from the spleen to the infected lung (9). Comparatively, the levels of IFN-γ^+^ or IL-2^+^ T cells, IFN-γ^+^ T_EM_, and IL-2^+^ T_CM_ cells in the lung of each group were much lower than that in the respective spleen. Compared with BCG-PBS control mice, repeat BCG vaccination induced a more significant increase in the number of IFN-γ^+^ or IL-2^+^ T cells, IFN-γ^+^ T_EM_, and IL-2^+^ T_CM_ cells in the lung (*p* < 0.05, Figure 7). Regardless of vaccine types (Figure S3B), heterologous boosters with CTT3H-based vaccines induced the most numbers of all these biomarkers, especially both IL-2^+^ T_CM_ and IFN-γ^+^ ${\text{CD}}_{4}^{+}$ T_EM_ cells than BCG-PBS control group as well as repeat BCG vaccination (Figure 7). In addition, IFN-γ^+^ ${\text{CD}}_{4}^{+}$ T cells and IFN-γ^+^ ${\text{CD}}_{4}^{+}$T_EM_ cells of all biomarkers were dominated in the lung of all BCG prime-CTT3H boost groups.

### Lung pathological lesion decreased by BCG prime-boosters against late persistent rather than primary TB infection

**Figure 8:**
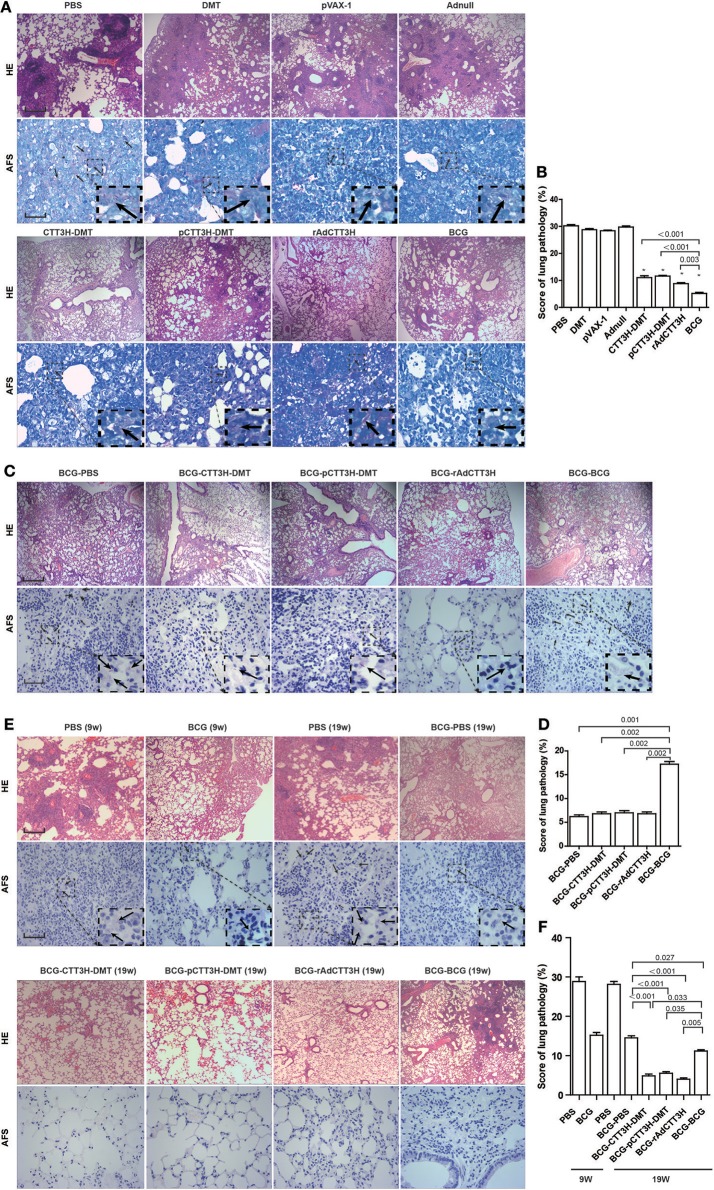
Comparison of histopathological changes in lungs among different regimens. The immunization and challenge schedules were shown in Figures 2A,D,G. Arrowheads indicate AF positive bacteria. HE, scale bar = 400 μm; AF staining, scale bar = 50 μm. **(A)** The representative lung pathological changes of three CTT3H-based vaccines alone immunized C57BL/6 mice against primary *M. tuberculosis* infection and **(B)** lung pathological scores (*n* = 3). **(C)** The representative lung pathological changes of BCG prime-CTT3H-based boosters in mice against primary infection and **(D)** lung pathological scores (*n* = 3). **(E)** The representative lung pathological changes of BCG prime-CTT3H-based boosters against mice late persistent TB infection and **(F)** lung pathological scores (*n* = 3).

As shown in Figure 8A, in the primary *M. tuberculosis* infection model, the most serious lung pathology with extensive fibrosis, perivasculitis, and alveolitis was obtained in all control groups, which also had the highest pathological score with acid-fast bacilli (AFB) throughout the whole lung tissue section. Compared to controls, three CTT3H-based vaccines displayed far fewer pathological lung changes with only a few AFB dispersed through the tissue section (Figure 8A). Consistent with the results of enumeration in organs, the lowest lung pathological score and the fewest AFB were found in the lung from BCG vaccinated mice among all groups (Figures 8A,B).

When pre-exposure boost BCG with CTT3H-based vaccines to against primary *M. tuberculosis* infection, consistent with the results of bacterial enumeration, more AFB stained bacteria were observed in the lung section from BCG repeat vaccinated mice, which also showed the most serious pathological changes (Figures 8C,D) with extensive fibrosis, perivasculitis, and alveolitis (Figures 8C,D). However, heterogeneous CTT3H boosters did not enhance the protection of the BCG vaccine against late persistent TB when compared to BCG-PBS control group. Interestingly, compared with BCG repeat vaccination, mice boosted with CTT3H-based vaccines or PBS resulted in fewer pathological changes and fewer AFB present in the lung (Figures 8C,D).

While post-exposure boost BCG with CTT3H-based vaccines to against persistent *M. tuberculosis* infection, among all groups, the most serious lung pathology with extensive fibrosis was found in the BCG-PBS group, and a few AFB were found in their lung tissue sections. Compared with the BCG-PBS group, lower lung pathological scores and fewer AFB in the lung tissue were obtained in CTT3H-based vaccines boosted mice (Figures 8E,F), consistent with the results of HE staining. Together with these data, heterologous post-exposure boost BCG with CTT3H-based vaccines significant improve lung pathological changes of late persistent *M. tuberculosi*s infected mice.

## Discussion

In this study, we compared the efficacy of different vaccine types, namely CTT3H-DMT, pCTT3H-DMT, and rAdCTT3H, as boosters to BCG vaccine-primed protection. In particular, immunogenicity and protection efficacy against primary TB infection and late persistent TB infection were evaluated. Our results demonstrated that similar protective efficacy was provided by CTT3H-DMT, pCTT3H-DMT, and rAdCTT3H alone in vaccinated mice against primary infection but that protection was inferior to that of the BCG vaccine. CTT3H-based vaccines as heterologous boosters did not enhance the protection primed by the BCG vaccine against primary infection, however, boost regimens conferred stronger protection against late persistent TB infection than BCG alone, regardless of vaccine types. Our findings demonstrate that a BCG prime-boost strategy might be a promising measure for the prevention against late persistent TB infection, rather than primary infection.

During past decades, BCG prime and heterologous boost strategy has been explored to protect adults against primary TB infection (6). Th1-biased response, especially increasing IFN-γ secreting CD4^+^T cells in the spleen of vaccinated mice were generally used as the biomarker to assess the immunogenicity of the strategy (5, 6). The majority of boosters (5, 6), as well as all heterologous boosters in this study, could elicit Th1-typed response, which cannot enhance the protection of the BCG vaccine against primary TB infection in mice. Therefore, it may not be a reliable biomarker to guide the development of booster vaccine for adult TB (16–18). In fact, cell-mediated immunity against *M. tuberculosis* always is slow to form when compared with other pulmonary pathogens such as *Influenza virus* (19) or *Streptococcus pneumoniae* (20), which benefits the replication of *M. tuberculosis* in the lung during the first 3 weeks after primary infection and subsequently promotes the establishment of a latent infection (21). IL-2^+^ T_CM_ cells are located in secondary lymphoid tissue. They are characterized by highly expressing IL-2 and can turn into effector T cells when they encounter the antigen again (22). T_EM_ cells are located in infection sites and can express IFN-γ very rapidly (22). Previously, we found that IFN-γ^+^ T_EM_ cells and IL-2^+^ T cells, especially IL-2^+^ T_CM_ cells in the spleen are related with the vaccine-induced protection (8). Further, sterilizing immunity plays a critical in the clearance of LTBI, which might be achieved by driving IFN-γ^+^ CD4^+^T_EM_ and IL-2^+^ T_CM_ cells to the infected lung (9). In this study, CTT3H-based boosters did not enhance the protection of the BCG vaccine against primary infection, which might contribute to the decreased levels of IFN-γ^+^ T_EM_ and IL-2^+^ T_CM_ cells in the spleen. A recent study also supported this conclusion that an improved protection conferred by an BCG prime-recombinant Sendai virus expressing antigen Ag85A and Ag85B boost regimen was related with the increasing levels of CD4^+^ T_CM_ cells (23). Furthermore, both IFN-γ^+^ T_EM_ cells and IL-2^+^ T_CM_ cells were increased significantly in the spleen and lung by post-exposure vaccination, regardless of BCG or CTT3H-based vaccines as boosters. Correspondingly, BCG primed mice boosted with BCG or CTT3H-based vaccines strongly inhibited the growth of persistent *M. tuberculosis* and provided more significant protection against late persistent TB infection than the BCG vaccine alone. The improved protection of all boosters was related with increasing number of IFN-γ^+^ T_EM_ cells and IL-2^+^ T_CM_ cells in the infected lung, consistent with our previous reports (8, 9). However, BCG re-vaccination or CTT3H based-boosters cannot thwart the reactivation as CMFO/DMT does (9). Our results were also supported by other previous reports. For example, T_CM_ cells are associated with LTBI, while T_EM_ cells are related with active TB (24). Moreover, an experiment of adoptive transferring of T cells from “immune” or vaccinated mice into immunodeficient recipient mice also demonstrated that central memory-like CD4^+^ T cells (CD62L^hi^) rather than effector memory T cells (CD62L^lo^) play an important role in the protection against TB (25). VPM1002, a modified BCG ΔureC::hly conferred superior protection against TB vs. BCG, which is also related with higher numbers of T_CM_ cells (26). In addition, mice chronically infected with *M. tuberculosis* could elicit higher number of T_CM_ cells than mice vaccinated with the BCG vaccine, which may contribute to the failure of the BCG vaccine to provide long-lasting protection (27). Taken together, IFN-γ^+^ T_EM_ cells, and especially IL-2^+^ T_CM_ cells and their distribution are more effective biomarkers to assess vaccine-induced protection, regardless of models of primary infection and late persistent infection.

In conclusion, the levels of IFN-γ^+^ T_EM_ cells and IL-2^+^ T_CM_ cells are related with vaccine-elicited protective efficacy and might be used as alternative biomarkers for management of LTBI or guiding preclinical and clinical trials of vaccine candidates. Antigens that can stimulate IL-2^+^ T_CM_ cell responses should be suitable targets for controlling of LTBI. For example, ESAT-6 might not be a suitable antigen candidate, because it induces ESAT-6-specific T cells driven toward terminal differentiation in persistent TB infections (28). Several subunit vaccine candidates, such as CMFO (9), ID93 (29), and H56 (30) might provide longer-lasting protection against primary infection than the BCG vaccine alone in different animal models. In the future, they could be developed to replace the BCG vaccine for the immunization of infants. Therefore, our results have important implications in guiding the clinical practice and future development of TB vaccine for adults.

## Ethics statement

The study protocols involving animal experiments were performed in accordance with the guidelines of the Chinese Council on Animal Care. The Committee on the Ethics of Animal Experiments and the School Committee on Biosafety of Tongji Medical College (Wuhan, China) approved the research protocols.

## Author contributions

YW and MC performed immunological detection, collection, and analysis of data. JM, XT, and MT performed the experiments with TB animal models. EB prepared the purification of antigen. XF designed the experiments, interpreted the data, and wrote the manuscript.

### Conflict of interest statement

The authors declare that the research was conducted in the absence of any commercial or financial relationships that could be construed as a potential conflict of interest.

## Supplementary material

Figure S1 TB10.4 CD8^+^ peptide-pulsed, CFSE-labeled target cells were analyzed for *in vivo* CTL activities from different immunized C57BL/6 mouse (*n* = 6). Representative histograms of the peptide-pulsed splenocyte targets with the right and left peaks being CFSE-labeled TB10.4 peptide-pulsed splenocytes and unpulsed splenocytes analyzed by a flow cytometer, respectively.

Figure S2 Comparison of CTT3H antigen-specific T cells in spleens between BCG prime-different CTT3H-based boosters before exposure (*n* = 6).

Figure S3 Comparison of CTT3H antigen-specific T cells in spleens and lungs between BCG prime-different CTT3H-based boosters after exposure (*n* = 6). Comparison of CTT3H antigen-specific T cells in spleens **(A)** and lungs **(B)** between BCG prime-different CTT3H-based boosters after exposure.
