# Supplementary figures and images for: Heterologous Boost Following Mycobacterium bovis BCG Reduces the Late Persistent, Rather Than the Early Stage of Intranasal Tuberculosis Challenge Infection
Source: Front Immunol. 2018 Oct 30;9:2439. doi: 10.3389/fimmu.2018.02439 (PMC6218689; doi:10.3389/fimmu.2018.02439)

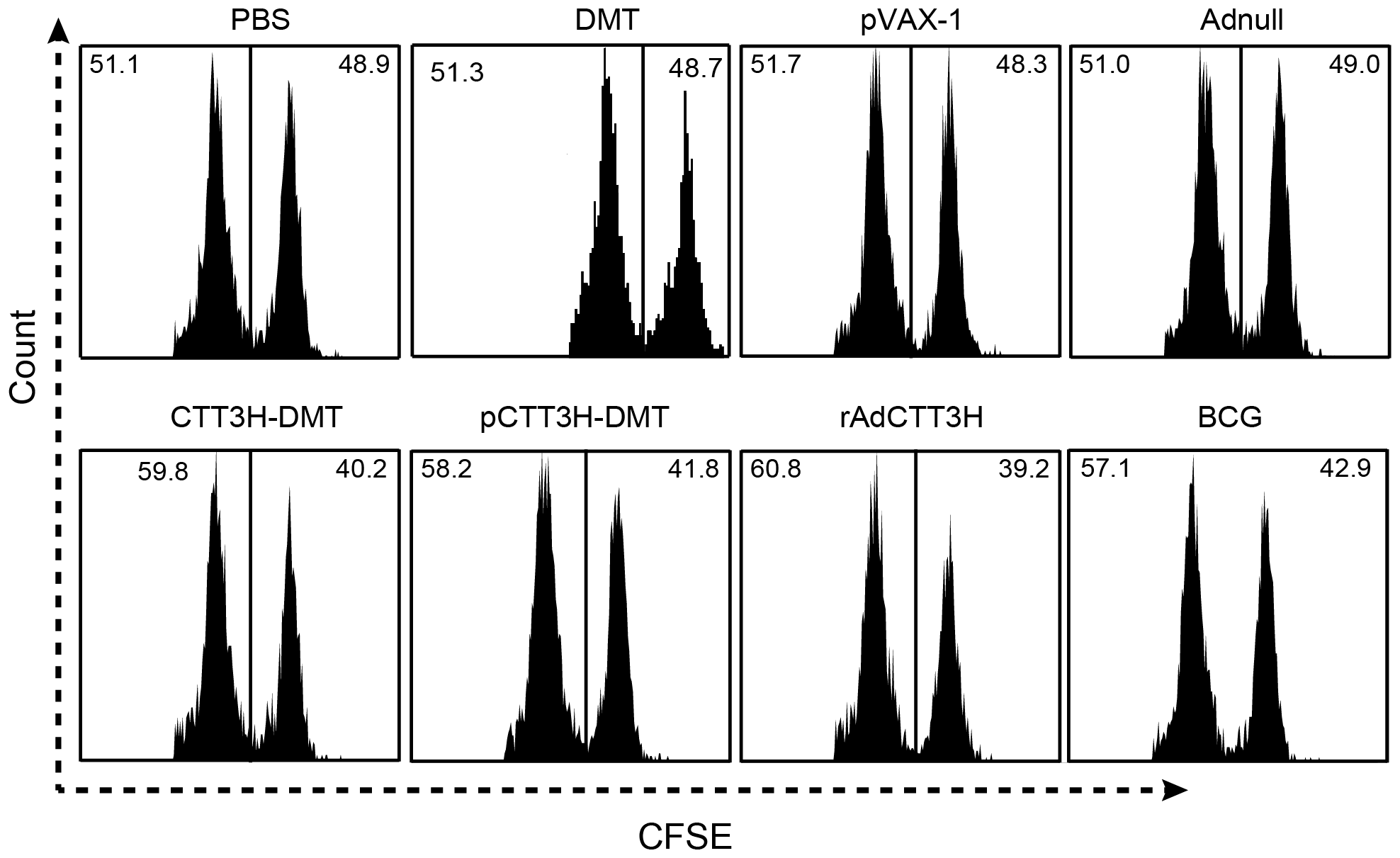

Supplement: Figure S1 — TB10.4 CD8+ peptide-pulsed, CFSE-labeled target cells were analyzed for in vivo CTL activities from different immunized C57BL/6 mouse (n = 6). Representative histograms of the peptide-pulsed splenocyte targets with the right and left peaks being CFSE-labeled TB10.4 peptide-pulsed splenocytes and unpulsed splenocytes analyzed by a flow cytometer, respectively. [file Image_1.TIF]

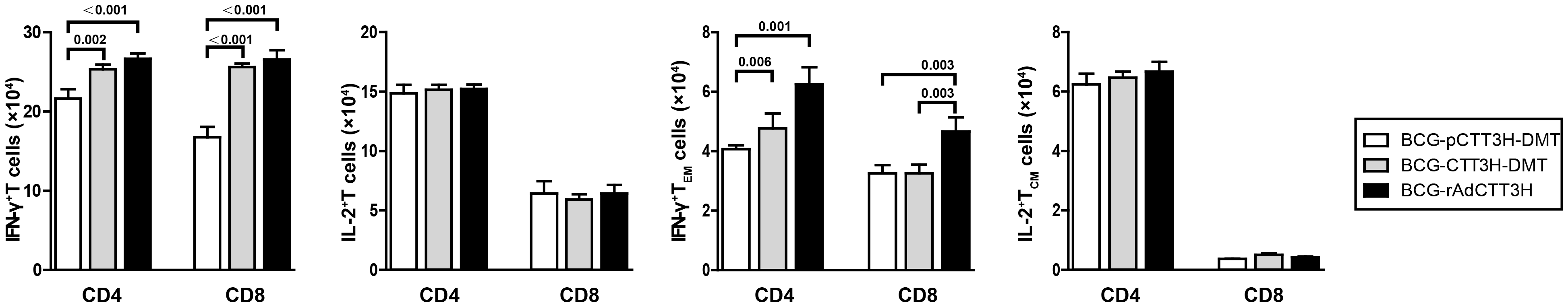

Supplement: Figure S2 — Comparison of CTT3H antigen-specific T cells in spleens between BCG prime-different CTT3H-based boosters before exposure (n = 6). [file Image_2.TIF]

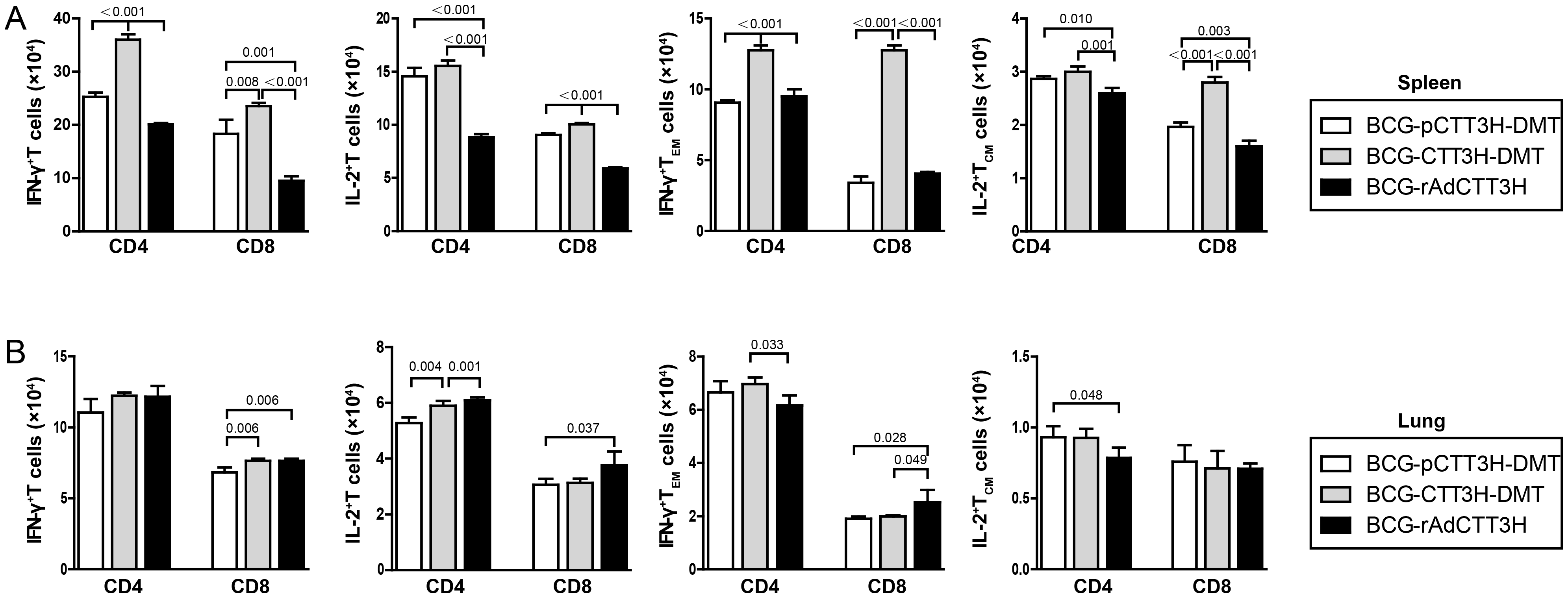

Supplement: Figure S3 — Comparison of CTT3H antigen-specific T cells in spleens and lungs between BCG prime-different CTT3H-based boosters after exposure (n = 6). Comparison of CTT3H antigen-specific T cells in spleens (A) and lungs (B) between BCG prime-different CTT3H-based boosters after exposure. [file Image_3.TIF]
